# Expression and clinical significance of NRLP1 in patients with ST-segment elevation myocardial infarction combined with malignant ventricular arrhythmia

**DOI:** 10.12669/pjms.39.4.7324

**Published:** 2023

**Authors:** SiXin Xie, HuiQiong Jiang, ZiLin Gao, YongJun Lin, NaJiao Hong

**Affiliations:** 1 SiXin Xie, Department of Cardiac Function Examination, First Hospital of Quanzhou Affiliated to Fujian Medical University, City Fujian Province, 362001, China; 2 HuiQiong Jiang, Department of Cardiac Function Examination, First Hospital of Quanzhou Affiliated to Fujian Medical University, City Fujian Province, 362001, China; 3 ZiLin Gao Department of Neurological rehabilitation, Quan Zhou Women’s and Children’s Hospital, City, Fujian Province, 362002, China; 4 YongJun Lin, Department of General Medicine, First Hospital of Quanzhou Affiliated to Fujian Medical University, City Fujian Province, 362001, China; 5 NaJiao Hong, Department of General Medicine, First Hospital of Quanzhou Affiliated to Fujian Medical University, City Fujian Province, 362001, China

**Keywords:** NRLP1, ST-segment elevation myocardial infarction, Malignant ventricular arrhythmia

## Abstract

**Objective:**

To investigate the clinical effects of NRLP1 expression in patients with ST-segment elevation myocardial infarction (STEMI) combined with arrhythmia.

**Methods:**

We enrolled 231 patients with STEMI in the first hospital of Quanzhou affiliated to Fujian Medical University from January 2019 to December 2020 to the observational group and 230 healthy individuals as the control group. We divided patients with STEMI into a malignant ventricular arrhythmia (MVA) group (*n*=36) and non-MVA(NMVA) group (*n*=195) depending on whether the individuals had experienced an episode of MVA within 48 hours after PCI. We recorded general variables such as age, gender, history of smoking, hypertension, of diabetes, hyperlipidemia, left ventricular ejection fraction (LVEF), Gensini score, and mortality. Moreover, we determined NLRP1, IL-1β, TNF-α, high-sensitivity C-reactive protein (hs-CRP), N-terminal pro-brain natriuretic peptide (NT-pro-BNP), cardiac troponin-1 (cTnI), and creatine kinase isoenzyme (CK-MB) in peripheral blood by ELISA.

**Results:**

We found significant differences in LVEF, Gensini scores, smoking history, and mortality between the MVA and NMVA groups. The mean NLRP1 expression was highest in the MVA group, which was positively correlated with the levels of IL-1β, TNF-α, hs-CRP, NT-pro-BNP, cTnI and CK-MB. The expression of NLRP1 was associated with the smoking history, the LVEF value, the Gensini score, the MVA incidence and the mortality. Patients with higher NLRP1 expression levels had a higher MACE incidence and worse overall survivals within one year.

**Conclusion:**

The NLRP1 pathway is associated with the presence of arrhythmias after PCI treatments, and the NLRP1 expression level may be useful as a predictor of arrhythmia in patients with STEMI.

## INTRODUCTION

ST-segment elevation myocardial infarction (STEMI) is a common cardiovascular disease, mostly resulting from thromboses caused by fracture of an unstable coronary plaque that leads to a complete coronary artery obstruction. Such an obstruction causes myocardial ischemia hypoxia and increases the risk of malignant ventricular arrhythmias (MVA).^1^ For the patient with STEMI, the main symptom is ischemic chest pain. Without a prompt and effective treatment, the patient’s life will be endangered as the disease progresses.^2^ The establishment of chest pain centers and the rapid development and popularization of percutaneous coronary intervention (PCI) technology has improved the survival rate of patients with STEMI.^3^ However, the prognosis of patients is influenced by many factors, and many complications are prone to occur.

MVA is one of the serious complications threatening the life of patients with acute STEMI. A multicenter randomized clinical controlled study identified baseline data and treatment variables as prognostic factors for patients with STEMI and arrhythmia.^4^ Early identification of high-risk patients and active preventive measures are important strategies to improve the prognosis of patients with STEMI and arrhythmias. Studies have shown that inflammatory factors are associated with the prognosis of patients with STEMI, especially in those with MVA.^5^,^6^ There are few reports on the association between MVA and inflammatory factors.

NLRP1 is a member of the NLR (Nod-like receptors) protein superfamily. It is involved in programmed cell death, and immune responses, and it is a major player of chronic inflammation.^7^,^8^ It has been shown that injury-inducing factors can be coupled to NLRP1 and recruit apoptosis-associated speck-like protein (ASC) and Pro-caspase-1, induce the activation of Pro-caspase precursors, lead to the release of inflammatory factors, and cause chronic inflammation.^9^ Inflammasomes are intracellular protein complexes that induce programmed cell death and activate inflammation at the same time. NLRP1 inflammasomes are involved in a variety of diseases, especially cardiovascular diseases,^10^ and NLRP1 expression has been closely associated with various diseases, such as atherosclerosis and myocardial ischemia/reperfusion (I/R) injury.^11^

Therefore, we aimed to investigate the clinical significance of NLRP1 expression in patients with STEMI; in particular, we compared the NLRP1 expressions in the serum of patients with STEMI with or without MVAs.

## METHODS

We enrolled 231 patients (observational group) with STEMI treated in the First Hospital of Quanzhou affiliated to Fujian Medical University from January 2019 to December 2020, and 230 (control group) healthy individuals who received electrocardiogram (ECG) testing to confirm the absence of cardiac disease during the same period (control group). We divided the 231 patients with STEMI into two subgroups according to whether they had developed MVA within 48 hours after PCI (MVA group; n=36) or not (NMVA group; n=195). The diagnoses of STEMI were made according to the definition of acute myocardial infarction (AMI) in the American Heart Disease Guidelines.^12^ The criteria we used to diagnose MVA included a Lown Grade-III or higher ventricular arrhythmia and sustained ventricular tachycardia, ventricular flutter, and ventricular fibrillation.

### Inclusion Criteria:

The inclusion criteria for study participants included being admitted to the hospital within 48 hours after AMI onset and meeting the above-mentioned diagnostic criteria. All patients received PCI.

### Exclusion Criteria:

We excluded patients with cardiac rupture, ventricular septal perforation, pulmonary embolism, aortic dissection, malignant tumor, immune system disease, systemic inflammation due to causes other than acute MI, taking anti-inflammatory drugs, and abnormal liver and kidney function from the study.

### Ethical Approval:

All patients signed informed consents, and the Ethics Committee of the First Hospital of Quanzhou Affiliated to the Fujian Medical University of Science and Technology (No. 2021-195, dated: 2021-01-01).

We collected general clinical information from all patients including age, gender, BMI, underlying disease, smoking status, left ventricular ejection fraction (LVEF), and Gensini score. In addition, we measured the serum expressions of NLRP1, IL-1β, IL-6, TNF-α, high-sensitivity C-reactive protein (hs-CRP), N-terminal pro-brain natriuretic peptide (NT-pro-BNP), cardiac troponin-1 (cTnI), and creatine kinase isoenzyme (CK-MB). Finally, we recorded major adverse cardiac events (MACE) after one-year follow-up, including recurrent angina, heart failure, re-myocardial infarction, or cardiac death after hospital discharge.

Blood samples of the patients were collected within 24 hours of admission and before the occurrence of arrhythmias. We used commercial enzyme-linked immunosorbent assay (ELISA) kits to detect the expressions of NLRP1 (Mybiosource, MBS924094), IL-1β (Boster, EK0392), IL-6 (Boster, EK0410), TNF-α (Boster, EK0525), hs-CRP (CUSABIO, CSB-E08617h), NT-pro-BNP (Abcam, ab263877), cTnI (Nanjing Jiancheng, H149-2), and CK-MB (Nanjing Jiancheng, H197-1) in the sera. We conducted follow-ups from the first day after PCI, and once a month after discharge. The follow-ups ended 12 months after the event. The follow-up methods included outpatient reviews, telephone or network software return visits, and others. We recorded any occurrences of MACE (recurrent angina pectoris, heart failure, re-myocardial infarction and cardiac death).

We applied Shapiro Wilk test to test the normality of the data (*P* > 0.05 indicated normal distribution). We expressed continuous variables as means ± SDs when normally distributed, and as medians (ranges) in other cases. We used Chi square tests for measurement data. Comparisons between the two groups of continuous data were accomplished using the Student t-test or the Mann-Whitney u-test. We plotted a Kaplan-Meier curve for analysis of the one year survival and applied Spearman’s analysis to determine the association between biological markers. We considered a *P*-value lower than 0.05 as statistically significant. All calculations were performed with SPSS 26.0.

## RESULTS

We found similar values for gender, age, BMI, and comorbidities (hypertension, diabetes) among all the subgroups of patients (including the patients in the control group). We found different mean values for hyperlipidemia, smoking history, and LVEF between the patients in the STEMI group and those in the control group (*P*<0.01), but the mortalities in all groups were similar. In addition, we found significant differences in smoking history, LVEF, Gensini score, and mortality between the patients in the MVA and NMVA groups (*P*<0.05; Table-I).

**Table-I:** Comparison of general data of patients.

	MVA group (n=36)	NMVA group (n=195)	Control group (n=230)	P1 (STEMI vs control)	P2 (MVA vs NMVA)
Age (years)	61.81±7.55	60.65±8.64	60.94±6.68	0.8745	0.4517
Gender (male: female) *n* (%)	19:17 (52.78:47.22)	109:86 (55.90:44.10)	110:120 (47.83:52.17)	0.1032	0.7294
BMI (kg/m^2^)	25.05±2.16	25.41±2.42	25.39±2.42	0.8776	0.4009
Hypertension *n* (%)	18 (50.00)	85 (43.59)	72 (31.30)	0.7317	0.4771
Diabetes mellitus *n* (%)	14 (38.89)	71 (36.41)	76 (33.04)	0.3980	0.7769
Hyperlipidemia *n* (%)	16 (44.44)	92 (47.18)	65 (28.26)	0.0000	0.7625
Smoking history *n* (%)	17 (47.22)	57 (29.23)	45 (19.57)	0.0022	0.0335
LVEF (%)	49.17±2.84	52.48±3.00	55.07±2.90	0.0000	0.0000
Gensini score	91.79±12.95	66.93±12.08	-	-	0.0000
Mortality *n* (%)	7 (19.44)	2 (1.03)	4 (1.74)	0.1619	0.0000

**Fig.1:**
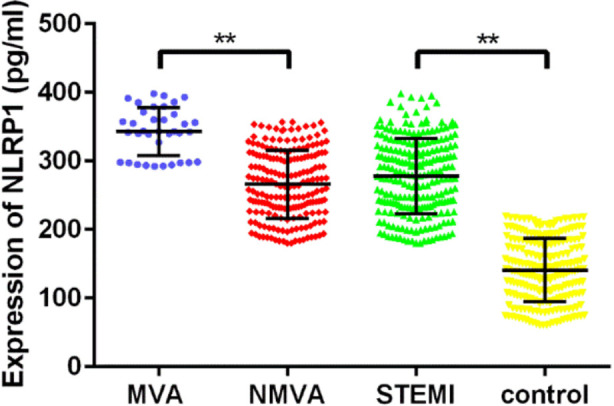
Expression of NLRP1 in serum of patients We used ELISA kits to detect the NLRP1 expression levels in the serum of patients from each group. **P<0.01

**Fig.2:**
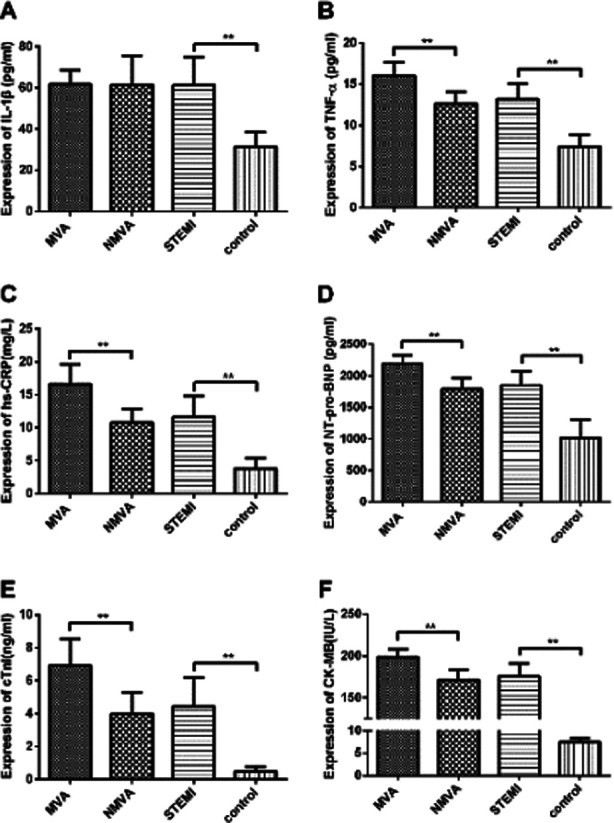
Expression of inflammatory factors and myocardial enzymes in patients. A: Mean serum IL-1β expression level for each group of patients. B: Mean serum TNF-α expression level for each group of patients. C: Mean serum hs-CRP expression level for each group of patients. D: Mean serum NT-pro-BNP expression level for each group of patients. E: Mean serum cTnI expression level for each group of patients. F: Mean serum CK-MB expression level for each group of patients. **P<0.01.

The mean ELISA NLRP1 expression levels in the serum for each group of patients is shown in Fig.1. The NLRP1 levels were 342.99 ± 34.78 pg/ml in the MVA group and 265.89 ± 49.54 pg/ml in the NMVA group for a significant difference (*P*<0.01). The mean NLRP1 level in the STEMI group (277.91±55.13 pg/ml) was significantly higher than that in the control group (140.68 ± 46.27 pg/ml; *P*<0.01). Considering the inflammatory pathway role of NLRP1, we detected the expression of other inflammatory factors (IL-1β, TNF-α and hs-CRP), and myocardial enzymes (NT-pro-BNP, cTnI and CK-MB) associated with STEMI (Fig.2) which shows that the serum levels of IL-1β, TNF-α, hs-CRP, NT-pro-BNP, cTnI and CK-MB in the STEMI group were significantly higher than those in control group (*P*<0.01).

The levels of TNF-α, hs-CRP, NT-pro-BNP, cTnI and CK-MB in the MVA group were significantly higher than those in the NMVA group (*P*<0.01). The levels of IL-1β were similar between the same groups. Table-II lists the results of our Pearson’s correlation analysis showing that IL-1β, TNF-α, hs-CRP, NT-pro-BNP, cTnI and CK-MB were positively correlated with the NLRP1 expression in patients with STEMI (*P*<0.01), the correlations were strongest for TNF-α, hs-CRP, NT-pro-BNP, cTnI and CK-MB.

**Table-II:** Correlations between the expressions of inflammatory factors and myocardial enzymes and NLRP1.

Variable	r	P
IL-1β	0.388	<0.01
TNF-α	0.701	<0.01
hs-CRP	0.672	<0.01
NT-pro-BNP	0.747	<0.01
cTnI	0.698	<0.01
CK-MB	0.746	<0.01

**Fig.3:**
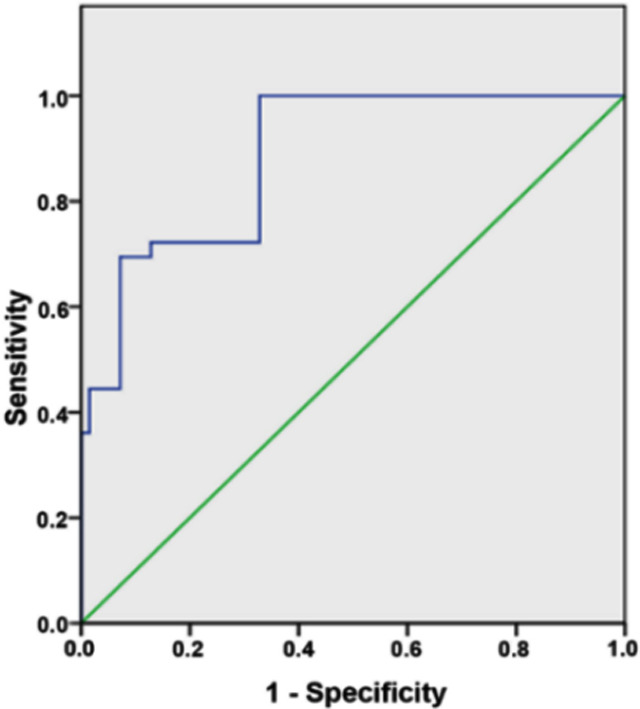
ROC curve to assess the role of NLRP1 in the diagnosis of MAV in patients with STEMI.

After dividing the data of patients according to the expression of serum NLRP1, we obtained a high NLRP1 expression group (*n*=116) and a low NLRP1 expression group (*n*=115). We then analyzed the association between the NLRP1 expression and clinicopathological variables in patients with STEMI. Table-III indicated that the NLRP1 expression was associated with smoking history, LVEF, Gensini score, MAV incidence and mortality (*P*<0.05), but not with age, gender, BMI, history of hypertension, diabetes or hyperlipidemia. To assess the diagnostic performance of NLRP1, the ROC curve analysis selected 292.2 pg/ml as the best cutoff value for NLRP1 (AUC=0.886; 95% CI: 0.835–0.937; sensitivity, 100.0%; specificity, 67.2%, *P*<0.001). Thus, the NLRP1 level should be useful to predict the occurrence of MVA in patients with STEMI. (Fig.3)

**Table-III:** Correlations between NLRP1 expression and clinic pathological variables of patients with STEMI.

	Cases	NLRP1 expression		P

		High (≥279.74pg/ml) *n*=116	Low (<279.74pg/ml) *n*=115	
Age (year)	231	59.92±7.51	61.74±9.28	0.1033
***Gender***				
Male	128	67 (57.76)	61 (53.04)	0.4710
Female	103	54 (46.55)	49 (42.61)	0.5466
BMI (kg/m^2^)	231	25.58±2.50	25.13±2.25	0.1453
Hypertension	103	55 (47.41)	48 (41.74)	0.3856
Diabetes mellitus	85	40 (34.48)	45 (39.13)	0.4639
Hyperlipidemia	108	61 (52.59)	47 (42.61)	0.1290
Smoking history	74	46 (39.66)	28 (24.35)	0.0127
LVEF (%)	231	51.17±3.25	52.77±2.97	0.0001
Gensini score	231	75.02±16.66	66.56±12.18	0.0000
Incidence of MVA (%)	36	36 (31.03)	0 (0)	0.0000
Mortality	9	8 (6.90)	1 (0.87)	0.0179

**Fig.4:**
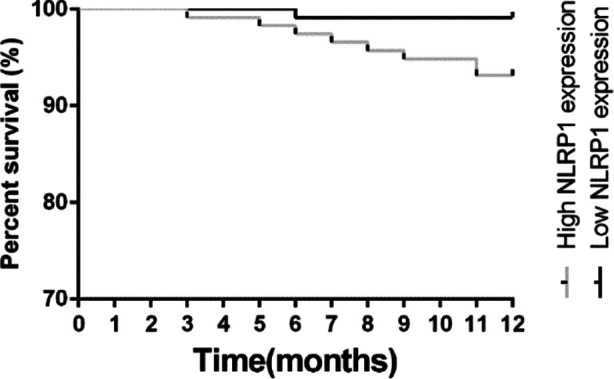
NLRP1 Kaplan-Meier curve and prognosis of patients with STEMI and MACE.

One-year follow-up data with MACE incidence recordings is shown in Table-IV. The incidences of MACE were 57.76%, in the high NLRP1 expression subgroup and 14.78% in the low expression group, suggesting that patients with higher NLRP1 expression levels have a higher incidence of MACE and a worse prognosis than those with low sNLRP1 expression levels. Moreover, the one year survival rate of the patients in the high NLRP1 expression group was markedly lower than that in the low NLRP1 expression group. Fig.4.

**Table-IV:** Comparison of MACE incidences between the two groups after one-year follow-up (cases, %).

	Recurrent angina pectoris	Heart failure	Re- myocardial infarction	Malignant arrhythmia	Cardiac death	Total incidence
High NLRP1 expression (n=116)	7 (6.03)	9 (7.76)	7 (6.03)	36 (31.03)	8 (6.90)	67 (57.76)
Low NLRP1 expression (n=115)	4 (3.48)	7 (6.09)	5 (4.35)	0 (0)	1 (0.87)	17 (14.78)
P	-	-	-		-	0.0000

## DISCUSSION

We investigated the clinical significance of NLRP1 expression in patients with STEMI complicated with arrhythmia. NLRP1 has become a research hotspot in various diseases. In a reperfusion mouse model and in hypoxia/reoxygenation (H/R)-treated primary mouse cardiomyocytes, Lei Cao et al. found that endoplasmic reticulum stress stimulates NLRP1 inflammasome activation by modulating NF-κB signaling activity, and that NLRP1 inactivation inhibits the cardiomyocyte injury induced by H/R.^11^ In a canine ischemia model, Wang M et al.^13^ reported that an increase in inflammation (caused by injection of IL-1) exacerbates ischemia-induced ventricular arrhythmias by modulating neuronal remodeling of the left stella ganglion. Neuroplasticity caused by inflammation may be a new mechanism and therapy for ventricular arrhythmias. These studies show that NLRP1 expression is associated with myocardial infarction occurrences. We speculate that NLRP1 expression may be used as an index to predict myocardial infarctions. Our results confirmed that the serum NLRP1 expression level of patients with MVA is high, and that the NLRP1 expression level is closely associated with the development of MVAs and with mortality. Patients with high NLRP1 expression had a higher incidence of MACE and a worse one-year survival time.

NLRP1 might be used as an index to predict MVAs in patients with STEMI. The pathogenesis of MVAs after PCI in patients with STEMI remains unclear; however, studies have shown involvement of several mechanisms including ischemia-reperfusion injury, myocardial metabolism and electrolyte disorders, myocardial autonomic changes (increased autonomic activity, reentry and abnormal trigger activity), and stress and inflammatory responses.^14^,^15^ Ventricular arrhythmias after AMI increase the mortality of patients six times, and the presence of MVA is thought to predict adverse clinical outcomes in patients with AMI.^16^

Therefore, identifying high-risk patients promptly is important to accurately assess their clinical prognosis and prevent MVAs. NT-pro-BNP, a neuroendocrine hormone is secreted when the myocardium is injured. The higher the level of NT-pro-BNP, the more serious a patient’s myocardial damage. Our results show that the plasma NT-pro-BNP levels in patients with STEMI and MVA were significantly higher than those in patients with STEMI without MVA. The cause of these high levels may be the rapid secretion of a large amount of NT-pro-BNP by cardiomyocytes released into the blood circulation in patients with MVA due to their increased ventricular load.^17^ NT-pro-BNP is closely related to myocardial damage in patients, and an increase in its level indicates an abnormal cardiac function and an increased risk of MVA.

Therefore, clinical detection of serum NT-pro-BNP levels can be used to predict the development of MVA. Our findings confirmed this, and we found that the NLRP1 level was positively correlated with the NT-pro-BNP level. Studies have shown that leukocytes are active during STEMI in the formation and maintenance of supraventricular arrhythmias, especially after atrial fibrillations and myocardial infarctions. Higher leukocyte levels reflect a larger myocardial infarction area.^18^,^19^ Hs-CRP is an inflammatory marker significantly associated with development of adverse cardiovascular events.^20^

A study found that the levels of hs-CRP were increased in patients with sudden cardiac death regardless of the presence or absence of intracoronary thrombosis, this suggests that the level of hs-CRP may be causally related to MVA deaths in patients with stable plaques.^21^ At the same time, hs-CRP is associated with the prognoses of patients with STEMI. During STEMI, inflammatory markers and thrombi acts as markers and trigger MVA. In our study, we confirmed that hs-CRP had a higher expression in the MVA group, and it was also positively correlated with the NLRP1 level. Perhaps this suggests that NLRP1 can be used as a predictor of MVA in patients with STEMI. NLRP1 can aggregate ASC under the action of exogenous injurious stimuli, it induces the activation of Caspase-1 protein precursor and promotes the production of IL-1β. IL-1β is essential for host responses and resistance to pathogens, but it also aggravates damage in chronic diseases and acute tissue injury.^11^

Anti-inflammatory therapy targeting interleukin IL-1β reduces the risk of recurrent cardiovascular events in patients with previous myocardial infarcts.^22^ The TNF-α levels have been reported to be elevated in the serum of patients with STEMI,^23^ a finding consistent with ours. Local injection of DNase-conjugated gold particles (AuNPs) into the rat myocardium can produce a 50% TNF-α knockout efficiency producing a significant anti-inflammatory effect, improving the acute cardiac function after myocardial infarction; therefore, TNF-α may be a potential treatment for myocardial infarction.^24^ On the basis of all the evidence, we believe that NLRP1 may affect the occurrence and prognosis of MVA in patients with STEMI by regulating the inflammatory response and many inflammatory factors.

### Limitations:

The study has limitations. As a single center study with retrospective data for analysis, the validation of the findings maybe attenuated. In addition, ROC cure was not be applied to predict MACE or death in MVA, and non-MVA group as well as in control group, which may provide more evidence for the study.

## CONCLUSION

The NLRP1 pathway is active during the process of arrhythmias after PCI treatments and the NLRP1 level may be used as a predictor of arrhythmias after PCI in patients with STEMI.

### Authors’ contributions:

**SX:** conceived and designed the study.

**HJ, ZG, YL and NH**: collected the data and performed the analysis.

**SX**: was involved in the writing of the manuscript and is responsible for the integrity of the study.

All authors have read and approved the final manuscript.
